# Extracellular vesicles secreted by *Saccharomyces cerevisiae* are involved in cell wall remodelling

**DOI:** 10.1038/s42003-019-0538-8

**Published:** 2019-08-09

**Authors:** Kening Zhao, Mark Bleackley, David Chisanga, Lahiru Gangoda, Pamali Fonseka, Michael Liem, Hina Kalra, Haidar Al Saffar, Shivakumar Keerthikumar, Ching-Seng Ang, Christopher G. Adda, Lanzhou Jiang, Kuok Yap, Ivan K. Poon, Peter Lock, Vincent Bulone, Marilyn Anderson, Suresh Mathivanan

**Affiliations:** 1 Department of Biochemistry and Genetics, La Trobe Institute for Molecular Science, La Trobe University, Melbourne, VIC 3086 Australia; 2 Department of Computer Science and Information Technology, La Trobe University, Melbourne, VIC 3086 Australia; 3 Cancer Research Division, Peter MacCallum Cancer Centre, Melbourne, VIC 3000 Australia; 4 Sir Peter MacCallum Department of Oncology, University of Melbourne, Melbourne, VIC 3010 Australia; 5 Bio21 Institute, University of Melbourne, Melbourne, VIC 3010 Australia; 6 ARC Centre of Excellence in Plant Cell Walls and Adelaide Glycomics, The University of Adelaide, Waite Campus, Urrbrae, SA 5064 Australia

**Keywords:** Proteomics, Biotechnology, ESCRT, Fungal biology

## Abstract

Extracellular vesicles (EVs) are membranous vesicles that are released by cells. In this study, the role of the Endosomal Sorting Complex Required for Transport (ESCRT) machinery in the biogenesis of yeast EVs was examined. Knockout of components of the ESCRT machinery altered the morphology and size of EVs as well as decreased the abundance of EVs. In contrast, strains with deletions in cell wall biosynthesis genes, produced more EVs than wildtype. Proteomic analysis highlighted the depletion of ESCRT components and enrichment of cell wall remodelling enzymes, glucan synthase subunit Fks1 and chitin synthase Chs3, in yeast EVs. Interestingly, EVs containing Fks1 and Chs3 rescued the yeast cells from antifungal molecules. However, EVs from *fks1*∆ or *chs3*∆ or the *vps23*∆*chs3*∆ double knockout strain were unable to rescue the yeast cells as compared to *vps23*∆ EVs. Overall, we have identified a potential role for yeast EVs in cell wall remodelling.

## Abstract (web summary)

Kening Zhao et al. show that yeast extracellular vesicles are depleted of ESCRT proteins but enriched with Fks1 and Chs3. The toxic effect of antifungal agents can be diminished by exposure to the Fks1- and Chs3- rich extracellular vesicles, suggesting a role for yeast extracellular vesicles in cell wall remodelling.

## Introduction

Extracellular vesicles (EVs) are released by cells under normal and disease conditions^1,2^. Secretion of EVs is thought to be conserved in both eukaryotes and prokaryotes, but has been best described for mammalian cells which release multiple EV subtypes designated as exosomes, ectosomes or shedding microvesicles and apoptotic bodies^3–5^. While exosomes are endocytically derived from the multivesicular bodies (MVBs) and are between 30 and 150 nm in diameter, ectosomes are shed from the outward budding of the plasma membrane and are between 100 and 1000 nm in diameter^6^. It is well established that the endosomal sorting complex required for transport (ESCRT) machinery is critical for the biogenesis of exosomes in mammalian cells^7,8^. Much of the knowledge on the function and interactions between the components of ESCRT machinery stems from yeast models^9^. Hence, due to the involvement in the biogenesis and their presence in EVs, human ESCRT components such as TSG101 and Alix are often used as markers or enriched proteins for EVs^5,10^. However, proteomic analyses of fungal EVs have not identified many ESCRT components as cargo. Though ESCRT deletion strains have been characterised to produce less EVs^11^, the effect of deletion of ESCRT components on EV cargo has not been investigated.

EVs have been isolated from a number of fungal species and are proposed to function in various host/pathogen interactions in both plants and animals^12,13^. In fungi, EVs are presumably involved in the transport of macromolecules across the fungal cell wall^12^. *Cryptococcus neoformans*, for example, produces secretory vesicles through a pathway involving MVBs. These vesicles are enriched with the polysaccharide glucuronoxylomannan which is incorporated into the capsule and delivered into host tissues as a virulence factor^14^. In addition, a number of proteins associated with virulence are also packaged in these vesicles^15^. EVs from *Paracoccidioides brasiliensis* and *Paracoccidioides lutzii* have been characterized with respect to their lipid^16^, proteome^17^, RNA^18^ and carbohydrate^19^ content. Interestingly, *P. brasiliensis* EVs induce an immune response in mice and promote M1 polarization of macrophages^20^. Similarly, vesicles produced by *Candida albicans* activate innate immune cells in vitro^21,22^. Antifungal drug resistance in *C. albicans* biofilms has also been linked to EV secretion^11^. Intracellular vesicles in *Saccharomyces cerevisiae* cells have been studied since the 1970s. Early work on yeast intracellular vesicles focused on a specific subclass of vesicles, termed chitosomes, which function in the biosynthesis of the cell wall polysaccharide chitin^23^. A more recent report described the composition of yeast EVs from wild type and mutant strains with defects in Golgi derived exocytosis or MVB formation^24^. All mutant strains produced EVs, but the proteomic profiles differed depending on the pathway that had been disrupted.

One major difference in secretion of EVs by fungi compared to mammalian cells involves the barrier, the fungal cell wall. A number of potential mechanisms for EV transit across the cell wall have been postulated^25^. From studies on the uptake of a liposomal formulation of the antifungal drug amphotericin B, it is clear that the cell wall has elastic properties^26^ that are possibly modulated by cell wall remodelling enzymes. Though fungal EVs have been reported to contain cell wall remodelling enzymes^12^, their role in cell wall remodelling has not been examined.

Here, we studied the role of the ESCRT machinery in the production of yeast EVs and examined the function of EVs in recipient cell wall remodelling. A panel of ESCRT knockout yeast strains was used to examine the role of the ESCRT pathway in EV production and composition. Label-free quantitative proteomics analysis revealed that yeast EVs are not enriched with ESCRT proteins as occurs with mammalian EVs. In addition, we discovered that yeast strains with defects in cell wall biosynthesis secrete more EVs than wild type (WT) cells. Further analysis revealed that yeast EVs containing the Fks1 and Chs3 proteins could rescue cells from the toxic effects of the antifungal agents, caspofungin and NaD1. These results demonstrate a previously undescribed cell wall remodelling property for EVs in fungal cells.

## Results

### Depletion of ESCRT components alters *S. cerevisiae* EVs

**Fig. 1:**
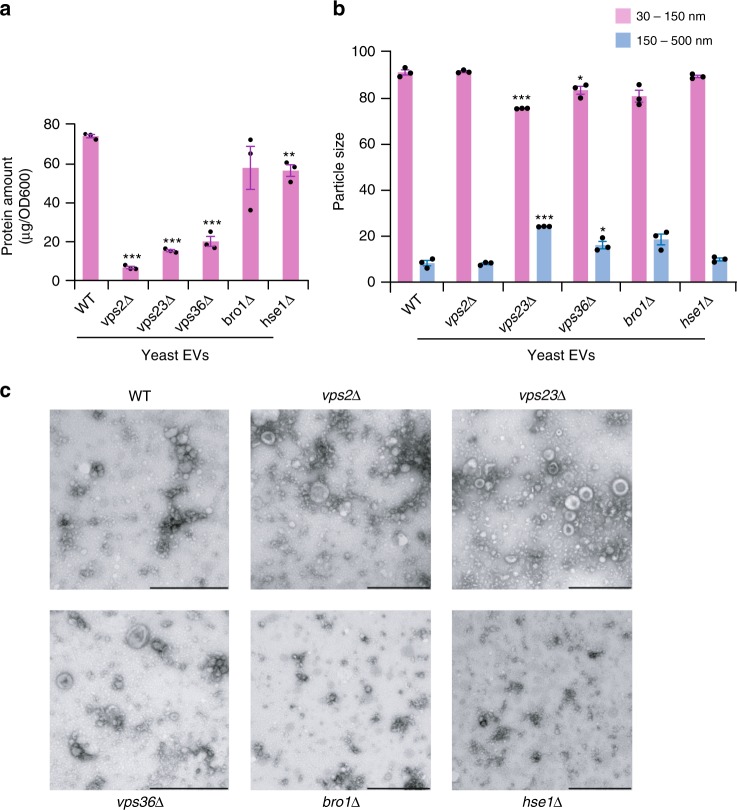
Characterization of yeast EVs by protein quantitation, NTA and TEM. **a** Yeast EVs were isolated from WT and ESCRT knockout strains. The isolated EVs were subjected to protein quantitation. Total protein amounts of EVs isolated from v*ps2∆, vps23∆* and *vps36*∆ were significantly less than EVs from WT (normalised to OD) (** denotes *P* ≤ 0.01, *** denotes *P* ≤ 0.001 as determined by two-tailed *t*-test; Error bar = ±SEM, *n* = 3 independent experiments). **b** NTA of EVs shows that vesicles between 150 and 500 nm diameter are enriched in *vps23*∆ and *vps36*∆ strains. On the contrary, vesicles between 30 and 150 nm diameter are depleted in *vps23*∆ and *vps36*∆ strains (* denotes *P* ≤ 0.05, *** denotes *P* ≤ 0.001; Error bar = ±SD, *n* = 3 independent experiments). **c** TEM analysis of EVs isolated from WT and ESCRT knockout strains shows that *vps23*∆ and *vps36*∆ yeast cells release bigger EVs. Scale bars; 500 nm

The role of the ESCRT machinery in the biogenesis of EVs was examined using a series of yeast knockout strains. The ESCRT machinery contains four complexes and accessory proteins. To understand the role of each ESCRT complex, one gene whose encoded protein is part of the complex was chosen for the yeast knockout strains. The deleted genes encoded single proteins in each of the four ESCRT subunits or the accessory proteins. They were Bro1 (ortholog of human Alix—ESCRT accessory proteins), Hse1 (ortholog of human STAM1 and 2—ESCRT 0), Vps23 (ortholog of human TSG101—ESCRT I), Vps36 (ortholog of human VPS36—ESCRT II) and Vps2 (ortholog of human CHMP2A and B—ESCRT III). EVs were isolated from WT and ESCRT knockout strains that had been grown for 18 h before the culture medium was collected and subjected to differential centrifugation coupled with ultracentrifugation. The total protein content and the morphology of isolated EVs was then examined. Strains with knockouts of the ESCRT components *vps2*Δ, *vps23*Δ and *vps36*Δ produced EVs with less protein than EVs from WT cells and strains with *hse1*Δ and *bro1*Δ knockouts (Fig. 1a). The morphological features and size of the EVs was examined using nanoparticle tracking analysis (NTA) and transmission electron microscopy (TEM) as recommended by MISEV standards^5,27^. NTA revealed a significant increase in the proportion of large EVs (150–500 nm) in the *vps23*Δ and *vps36*Δ knockouts (Fig. 1b). The reduction in EV release upon knockout of *vps2*Δ, *vps23*Δ and *vps36*Δ was further confirmed by NTA analysis (Supplementary Fig. 1a). Consistent with NTA results, TEM analysis revealed large vesicles in *vps23*Δ and *vps36*Δ knockouts (Fig. 1c). However, there was no significant difference in the size of EVs upon deletion of Vps2, Bro1 or Hse1. To ensure that the particles detected were EVs secreted from live cells, the isolation procedure was repeated with WT cells that had been heat killed. Very few particles between 30 and 150 nm were detected in the 100k pellet from heat-killed cells (Supplementary Fig. 1b) confirming that the EVs isolated from the growing cultures were not simply fragments of dead cells.

### Proteomic analysis reveals the depletion of ESCRT proteins

**Fig. 2:**
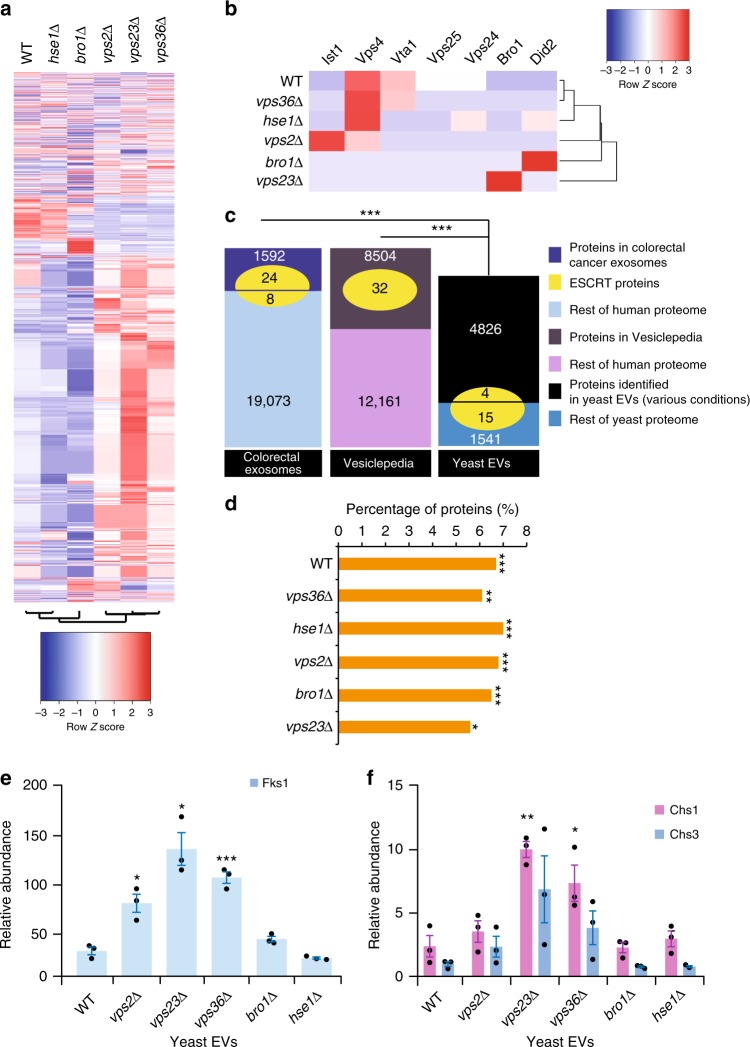
Label-free quantitative proteomics analysis of yeast EVs. **a** Heatmap depicting the proteomic profile (*n* = 3 independent experiments) of EVs isolated from WT and ESCRT knockout yeast cells. WT and *hse1*∆ yeast cell EV proteomic profile clustered together while *vps*∆ yeast cell EVs clustered together. **b** Heatmap of ESCRT subunits identified in yeast EVs. The detected ESCRT subunits were not enriched in any of the yeast EV samples. **c** Venn diagram depicting the overlap of ESCRT subunits with proteins identified in the EVs from colorectal cancer, Vesiclepedia and yeast. Each dataset is broken as proteins that were detected in EVs and proteins that are part of the entire proteome but were not detected in EVs. The ESCRT subunits were queried to identify if they were detected in the EV or the non-EV fraction. Compared to the human EV dataset, WT yeast EVs are significantly depleted of ESCRT components. *** denotes *P* ≤ 0.001 as determined by Chi-square test. **d** Proteins with AAA domains that are important for membrane fusion are enriched in yeast EVs compared to the entire proteome (* denotes *P* ≤ 0.05, *** denotes *P* ≤ 0.001 as determined by hypergeometric test in FunRich^28^). **e**, **f** Functional enrichment analysis of EV proteins highlighted that the EVs are enriched with proteins implicated in cell wall remodelling. Fks1 is enriched in *vps2*Δ, *vps23*Δ and *vps36*Δ EVs while Chs1 was enriched in *vps23*Δ and *vps36*Δ EVs. * denotes *P* ≤ 0.05, ** denotes *P* ≤ 0.01, *** denotes *P* ≤ 0.001 as determined by two-tailed *t*-test; Error bar = ±SEM, *n* = 3 independent experiments

The protein cargo of EVs from WT and knockout yeast strains was identified using an LC-MS/MS-based label-free quantitative proteomics analysis. Equal amounts of protein (30 μg) from the isolated EVs were subjected to SDS-PAGE and separated proteins were excised from the gel, reduced, alkylated and digested with trypsin. At an FDR of <1%, a total of 3133 proteins were identified in the yeast EVs (Supplementary Data 1; data deposited to Vesiclepedia), which represents a remarkable 52% coverage of the total yeast proteome. A heatmap (FunRich^28^) of the proteomic profile of the isolated EVs revealed clustering between *vps2*Δ, *vps23*Δ and *vps36*Δ strains while EVs from WT, *hse1*Δ and *bro1*Δ were grouped together (Fig. 2a). Hence, the proteomic analysis confirmed that knockout of the ESCRT components altered the protein cargo of the EVs.

Surprisingly, a follow up interrogation of the proteomic results highlighted the depletion of ESCRT components in yeast EVs (Fig. 2b, c). To calculate the statistical significance of this observation, EV proteomic datasets were downloaded from Vesiclepedia^29,30^ using FunRich. For an individual human colorectal cancer-derived EV dataset, 24 ESCRT components were identified among the 1592 EV proteins. Similarly, all the known 32 ESCRT components were detected in Vesiclepedia (mammalian) among a total of 8504 proteins reported in EVs. Compared to the colorectal cancer EV dataset and Vesiclepedia, EVs from WT yeast were depleted in ESCRT components. Next, a domain enrichment analysis using FunRich was performed on the proteins identified in EVs. Even though the yeast EVs were depleted with ESCRT components, they were enriched with proteins containing AAA domains that are implicated in membrane fusion^31–33^ (Fig. 2d). Furthermore, EVs from Vps knockout yeast strains were enriched with fungal cell wall remodelling enzymes including Fks1 (Fig. 2e), Chs1 and Chs3 (Fig. 2f). Chitin synthases Chs1 and Chs3 were enriched in EVs from *vps23*Δ and *vps36*Δ while the catalytic subunit of the major 1,3-β-glucan synthase Fks1 was enriched in EVs from *vps2*Δ, *vps23*Δ and *vps36*Δ. Overall, the proteomic analysis highlighted that the yeast EVs may be distinct from mammalians EVs in terms of their enriched proteins and physiological function. Furthermore, the enrichment of cell wall remodelling factors suggest that yeast EVs could be involved in regulating the dynamics of the yeast cell wall. To address these possibilities, the origins, as well as functions of yeast EVs, were investigated further.

### Cell wall mutants secrete more EVs

EVs have been proposed to function in the transport of a variety of cellular cargo across the fungal cell wall^12^. In this scenario, the cell wall acts as a barrier to EV release and uptake. This led to the hypothesis that yeast mutant strains with weakened cell walls would secrete more EVs into the culture medium. To assess this, EVs were isolated from strains with deletions in the major cell wall chitin synthase and 1,3-β-glucan synthase subunit, *chs3*Δ and *fks1*Δ, respectively. Protein levels in the EV fractions from these two strains was significantly higher than in WT, suggesting that the strains with defects in cell wall biosynthesis were releasing more EVs (Supplementary Fig. 2a). The relative increase in EV release from each polysaccharide synthase mutant correlates with the abundance of the respective polysaccharide in the yeast cell wall. That is, *fks1Δ* which functions in synthesis of 1,3 β-glucan (~50% dry weight of cell wall) released more EVs than *chs3Δ* which functions in synthesis of chitin (1–2% dry weight of cell wall)^34^. This indicates that the cell wall is likely acting as a barrier to EV release because EV release increases when the barrier is weakened.

### Subtypes of EVs produced by *S. cerevisiae*

**Fig. 3:**
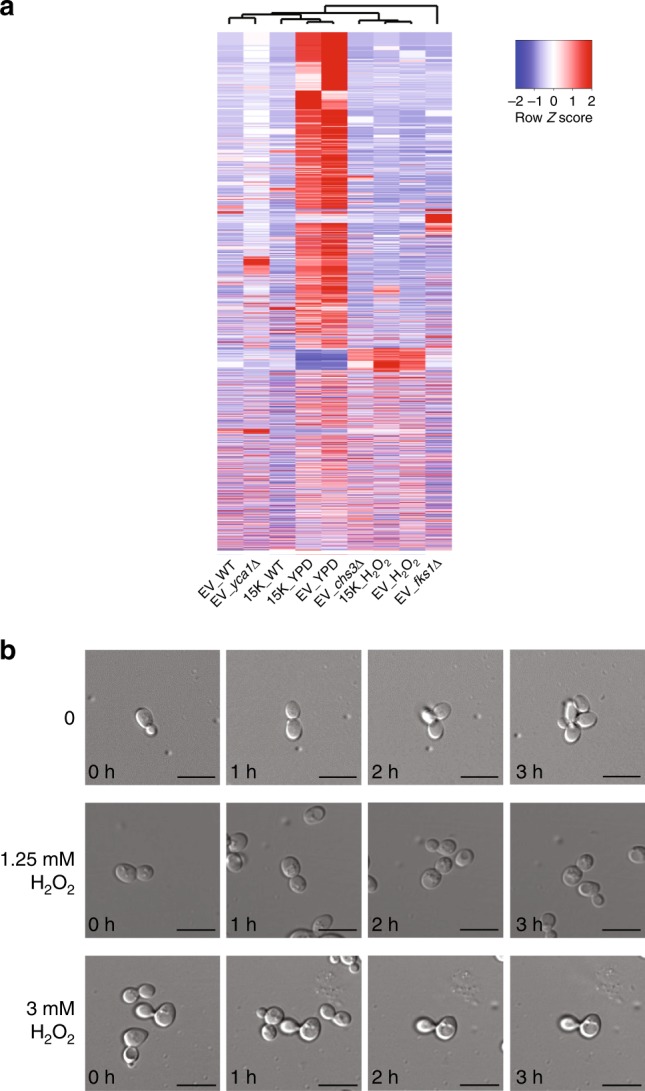
Proteomic analysis and live imaging of EVs isolated from yeast that had been perturbed with EV subtype inducers and inhibitors. **a** Isolated samples were subjected to label-free quantitative proteomics analysis. Proteomic profiles of WT EVs differed to other EV samples. **b** Live imaging of 1.25 mM and 3 mM H_2_O_2_ treated yeast cells did not show any release of apoptotic bodies. Scale bars; 10 μm

EVs from mammalian cells can originate endocytically (exosomes) or via membrane blebbing (ectosomes or microvesicles) or through apoptosis (apoptotic bodies)^4^. To understand the subtype of EVs secreted by WT yeast cells, a comparative analysis was performed. The secretion of EV subtypes was assessed using mutants and chemical inhibitors that distinguish between the different pathways employed for EV production in mammalian cells. WT yeast strains were treated with H_2_O_2_ that induces apoptosis. Prior to EV collection, tolerance tests for H_2_O_2_ were performed to establish suitable concentrations for treatment of the yeast cultures (Supplementary Fig. 2). A knockout strain of Yca1, a metacaspase required for apoptosis in yeast, was also used. EVs were isolated from WT cells that had been treated with H_2_O_2_ as well as the mutant strains (*chs3*Δ, *fks1*Δ and *yca1*Δ) (Supplementary Data 2; data deposited to Vesiclepedia). Larger EVs were also isolated from the WT culture by centrifugation at 15,000 × *g*. The isolated EV samples (Table 1) along with whole-cell lysates were subjected to label-free quantitative proteomic analysis. Heatmap based clustering of the proteomic profiles (5533 proteins – 92% of yeast proteome) revealed that EVs from WT cells were different to other EV samples (Fig. 3a). To determine whether apoptotic body-like vesicles are produced by yeast cells, time-lapse live imaging was employed. No apoptosis were detected upon treatment with H_2_O_2_ at 1.25 or 3 mM. The lack of cell breakage and vesicle release indicates that apoptotic bodies are not formed during yeast programmed cell death (Fig. 3b). Hence, from the EV subtype enrichment and proteomics analysis, it can be concluded that the yeast EVs are heterogeneous with different proteomic profiles.

**Table 1:** List of yeast EV subtypes

	Test	Control
100K pellet (EV_)	H_2_O_2_ (1.25 mM) treated	WT
	yca1	
	chs3	YPD media (equal volume with WT)
	fks1	
15K pellet (15K_)	H_2_O_2_ (1.25 mM) treated	WT YPD media (equal volume with WT)

### EVs enhances cell viability upon cell wall stress

**Fig. 4:**
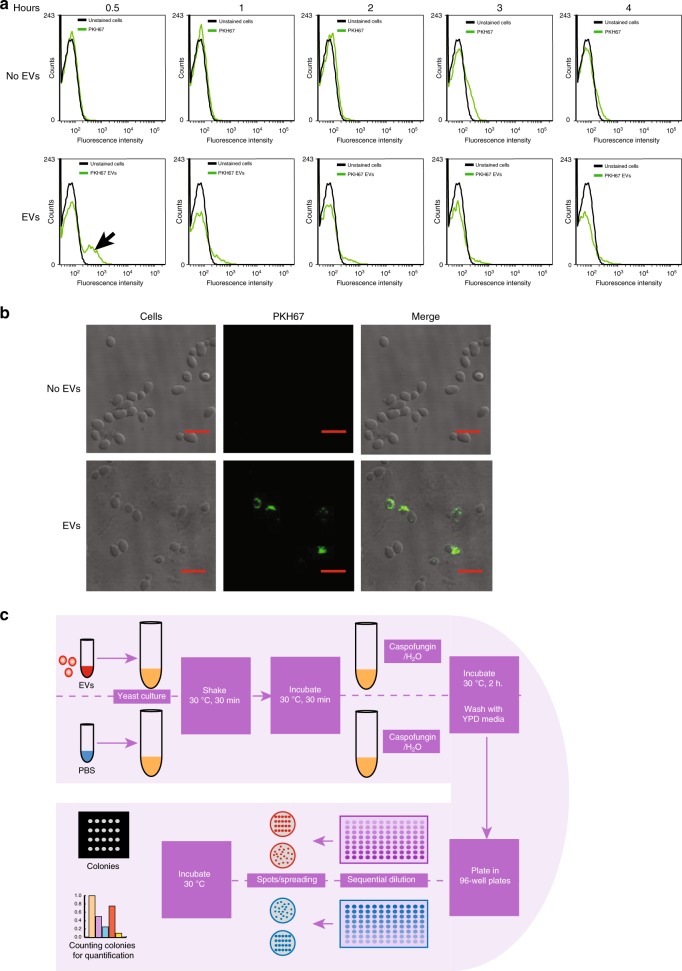
Uptake of EVs by yeast cells by FACS and confocal microscopy. **a** WT EVs (100 µg) were labelled with green fluorescent dye PKH67, subjected to washing and ultracentrifugation and incubated together with WT cells for 0.5, 1, 2, 3 and 4 h at 30 °C. FACS screening revealed an increase in fluorescent cells after 30 min of incubation. Cells interacting with PKH67 dye (with or without EVs) is depicted by green peak. Black peak represents the background fluorescence from control cells. ‘No EVs’ represent PKH67 dye alone control while ‘EVs’ represent PKH67 labelled EVs (*n* = 3 independent experiments). **b** Confocal microscopy revealed fluorescence within cells after 30 min of incubation. ‘No EVs’ represent PKH67 dye alone control while ‘EVs’ represent PKH67 labelled EVs (*n* = 3 independent experiments). Scale bars; 10 μm. **c** A schematic illustration of the functional uptake assay used to study the cell wall remodelling functions of EVs. EV concentration: 10 µL of 1 µg/µL EVs + 10 µL of OD 0.1 yeast + 80 µL YPD

It was evident from the proteomic analysis that EVs are enriched with cell wall remodelling enzymes including Chs3 and Fks1. Furthermore, knockout of *vps23* and *vps2* results in enrichment of Chs3 and Fks1 in the EVs. This led to the hypothesis that EVs could contribute to cell wall remodelling. To examine whether EVs can be taken up by yeast cells, we optimized an EV uptake assay using FACS and confocal microscopy. WT EVs were labelled with the green fluorescent lipophilic dye PKH67 and these tagged EVs were added to yeast cells and incubated with shaking at 30 °C for different periods of time. As shown in Fig. 4a, FACS screening revealed an increase of fluorescent cells after 30 min of EV incubation. This was confirmed by confocal microscopy which revealed fluorescence within cells after the 30 min incubation (Fig. 4b). These results confirm that EVs can be taken up by yeast cells.

**Fig. 5:**
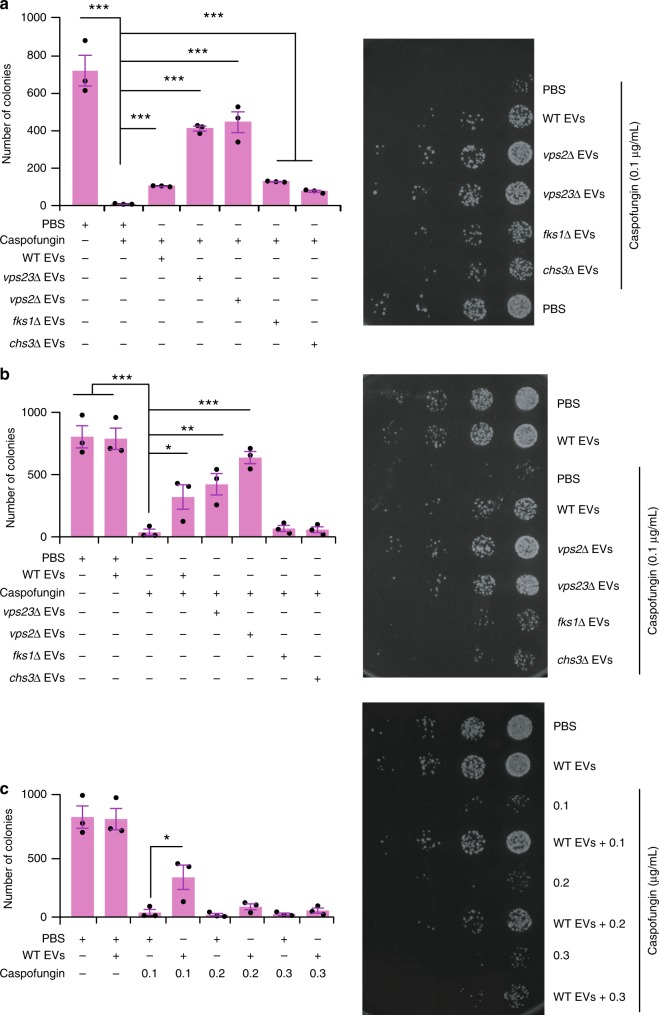
Functional uptake assay of EVs revealed protection against antifungal agents. **a** The functional uptake assay with the *chs3*Δ strain revealed that more yeast cells survived exposure to antifungal agents after pre-treatment with EVs. The EVs from five strains with knockouts in ESCRT genes were tested, but EVs from *vps2*Δ and *vps23*Δ cells provided the best protection. EV concentration: 10 µL of 1 µg/µL EVs + 10 µL of OD 0.1 yeast + 80 µL YPD. **b** The functional uptake assay with the WT strain revealed the best survival when the cells had been pre-incubated with EVs from WT, *vps2*Δ and *vps23*Δ cells. In contrast, EVs from *fks1*Δ and *chs3*Δ cells did not exhibit significant rescue. **c** The functional uptake assay with the WT strain and increasing concentrations of caspofungin. EV rescue was significant at 0.1 µg/mL caspofungin, but was not significant with 0.2 µg/mL and 0.3 µg/mL concentrations. Error bar for all data is presented as ± SEM; * denotes *P* ≤ 0.05, ** denotes *P* ≤ 0.01, *** denotes *P* ≤ 0.001 as determined by two-tailed t-test; *n* = 3 independent experiments

Next, an assay was performed to assess the cell wall remodelling properties of EVs (Fig. 4c). This was conducted using a recipient yeast strain with defective cell wall (*chs3*∆). These cells are more sensitive to the 1,3-β-glucan synthase inhibitor, caspofungin, because reduced chitin synthase activity makes them more dependent on 1,3-β-glucan for cell wall integrity^35^. EVs from WT and mutant yeast cells were tested for their ability to protect the *chs3*∆ cells from the deleterious effects of caspofungin. Before these tests were initiated, a tolerance test for caspofungin was performed to determine a suitable concentration (0.1 µg/mL) for treating WT and *chs3*∆ yeast cells. EV uptake assays were then performed where the *chs3∆* yeast strain was incubated with EVs from WT, *vps23*Δ, *vps2*Δ, *fks1*Δ and *chs3*Δ strains for 60 min at 30 °C to allow uptake of the EVs and time for the cargo to elicit its function. Next, the yeast culture was treated with caspofungin for 2 h. Cultures were washed and plated to visualize changes in cell viability in response to EV uptake and/or caspofungin treatment. EVs from *vps2*Δ and *vps23*Δ cells were most effective at rescuing caspofungin treated *chs3*∆ cells. WT, *fks1*∆ and *chs3*∆ EVs also had some protective effect, but to a lesser extent than the *vps2*Δ or *vps23*Δ EVs (Fig. 5a). Similarly, WT yeast strains were treated with EVs to determine whether resistance to caspofungin was enhanced in normal cells. An additional WT EV alone control was used in this assay to establish that EVs do not alter the cell viability. Consistent with previous results, EVs from *vps2*Δ*, vps23*Δ and WT protected the WT yeast strain against the antifungal activity of caspofungin and the *fks1*∆ and *chs3*∆ EVs did not rescue the WT yeast strains from caspofungin (Fig. 5b).

**Fig. 6:**
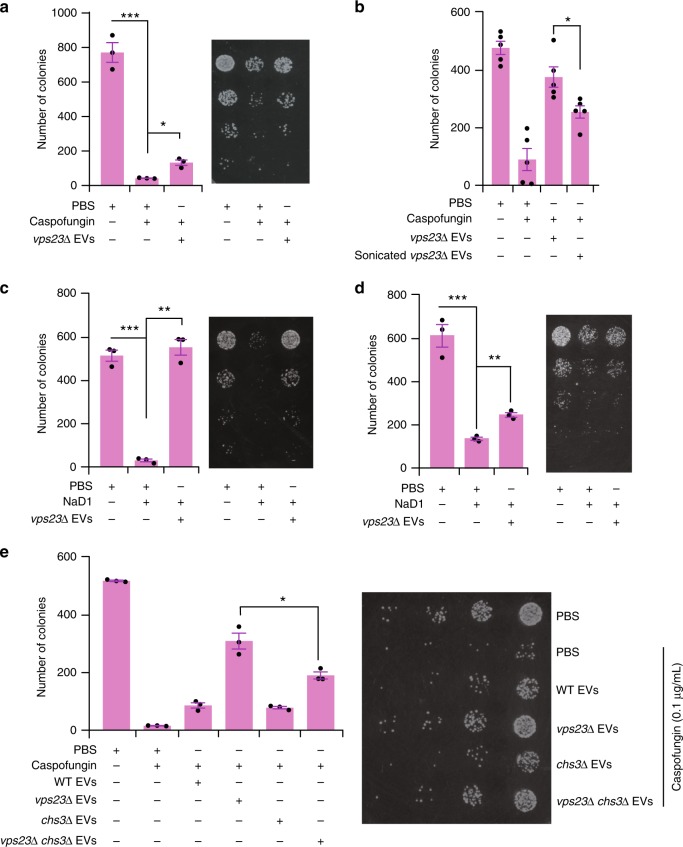
Chs3 containing EVs rescue yeasts from antifungal agents. **a** The rescuing effect of *vps23*Δ EVs was retained in the functional uptake assay that had been modified by an additional washing step before caspofungin (0.1 µg/mL) treatment. **b** Functional uptake assay revealed a decreased rescuing effect of sonicated vps23Δ EVs compared to unsonicated vps23Δ EVs (* denotes *P* ≤ 0.05, Error bar = ±SEM, *n* = 5). **c** A functional uptake assay with NaD1 (3 µM) treatment instead of caspofungin treatment revealed the consistent rescuing effect of *vps23*Δ EVs. **d** NaD1 (3 µM) functional uptake assay with an additional washing step before caspofungin treatment shows the retained rescuing effect of *vps23*Δ EVs. **e** Functional uptake assay revealed a decreased rescuing effect of *vps23*Δ*chs3*Δ EVs compared to *vps23*Δ EVs. Error bar for all data is presented as ±SEM; * denotes *P* ≤ 0.05, ** denotes *P* ≤ 0.01, *** denotes *P* ≤ 0.001 as determined by two-tailed *t*-test; *n* = 3 independent experiments. Caspofungin concentration used in this figure is 0.1 µg/mL

To understand the rescuing efficiency of EVs, the experiment was repeated with increasing concentrations of caspofungin. EVs protected cells against up to 0.1 µg/mL caspofungin, but the effect of the EVs was overwhelmed by caspofungin at 0.2 and 0.3 µg/mL. In the absence of higher concentration of caspofungin, there was no significant difference in survival between the EV treated and untreated cells (Fig. 5c). This indicates that the protective effect of EVs is dependent on the dose of the antifungal agent. We considered whether this protective effect had resulted from direct binding of the caspofungin to the EVs decreasing access to the glucan synthase in the plasma membrane or substantial uptake of the EVs and efficient cell wall reinforcement. As shown in Fig. 5b, EVs from the *fks1*∆ and *chs3*∆ strains did not rescue the WT yeast cells from caspofungin, indicating that it is not merely the caspofungin binding to the membrane of EVs, but it is the cargo that determines their protective effect. To investigate whether uptake of the EVs is required for protection against caspofungin, an additional thorough wash of the WT yeast cells was performed after the cells had been incubated with the EVs for 1 h and before the addition of caspofungin (0.1 µg/mL). The *vps23*∆ EVs still significantly increased the survival of WT yeast, even when free EVs had been removed from the system (Fig. 6a). To confirm that the rescuing effect of EVs is dependent on intact vesicles carrying cargo and not an impurity that co-purified with the EVs during isolation, sonication was performed to disrupt the intact membrane structure of EVs. Sonicated *vps23*Δ EVs provided less protective activity compared to unsonicated *vps23*∆ EVs (Fig. 6b), indicating that intact membranes of EVs are crucial for protective effect against the cell wall targeting antifungal caspofungin. These observations confirm that the rescue is mediated, at least in part, by delivery of the EV cargo to the recipient cells. Furthermore, to determine whether the protective effect of EVs was specific to caspofungin or was a general protective mechanism, *vps23*Δ EVs were tested for their ability to protect yeast cells against the antifungal plant-defensin, NaD1. Consistent with the caspofungin treatment, *vps23*∆ EVs also increased the survival of WT yeast cells after challenge with NaD1 (3 µM; Fig. 6c), even when the additional thorough wash had been applied (Fig. 6d). Hence, these data indicate that EVs protect yeast cells against antifungal agents that target the cell wall. Whether the mechanism of protection is the same across various antifungals remains to be elucidated.

### Chs3 containing EVs enhance cell viability

From previous proteomic analyses, it was evident that the *vps23*∆ and *vps2*∆ yeast cells secreted high amounts of cell wall remodelling enzymes including Chs3 via EVs (Fig. 2f). Furthermore, EVs from *chs3*∆ cells failed to rescue WT cells from the toxic effects of caspofungin (Fig. 5b). To understand whether EV-associated Chs3 regulates the mechanism of cell protection against antifungal agents, a double knockout, yeast strain of Vps23 and Chs3 was established. The *vps23*Δ*chs3*Δ EVs were then isolated for functional uptake assays. Consistent with previous results, EVs from WT and *vps23*∆ yeast cells increased the WT cell viability in presence of caspofungin. Interestingly, the rescue effect of *vps23*Δ*chs3*Δ EVs was significantly weaker compared to *vps23*∆ EVs (Fig. 6e), suggesting it is the elevated levels of Chs3 in EVs from the *vps23*∆ mutant that is responsible, at least in part, for the rescue properties of these EVs. However, the rescuing efficiency was not completely abolished in the double knockout suggesting that other cargo molecules also aid in the protection against these antifungals. Nevertheless, these results support the notion that uptake of Chs3 via EVs has a critical role in cell wall remodelling and that the EV cargo is responsible for the protective effect of EVs against antifungals.

### Caspofungin treatment increases EV release in *S. cerevisiae*

**Fig. 7:**
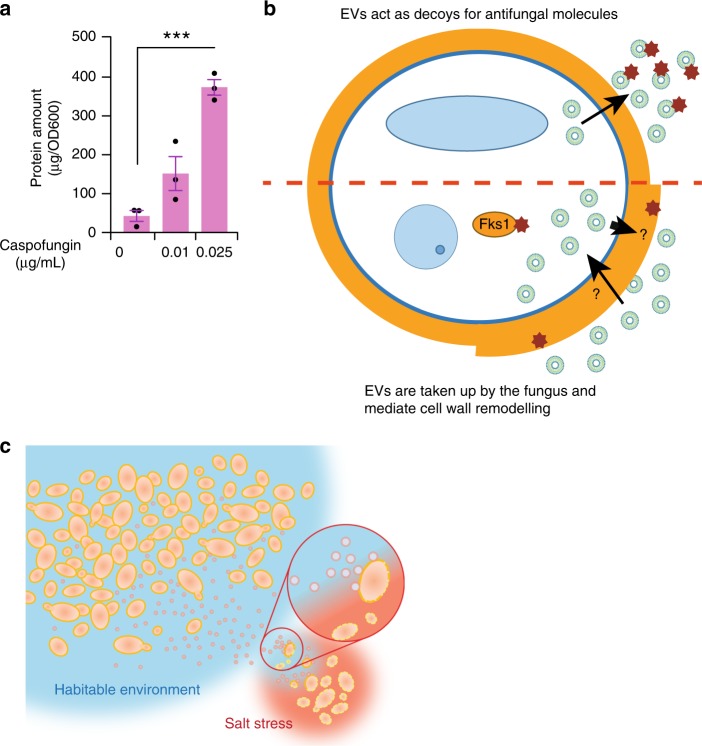
Caspofungin treatment increases the EVs secreted by yeast cells. **a** Total protein amounts of EVs isolated from 0.025 µg/mL caspofungin treated WT yeast were significantly more than untreated WT (normalized to OD, *** denotes *P* ≤ 0.001, Error bar = ±SEM, *n* = 3 independent experiments). **b** A graphical representation to depict the two potential roles of EVs in protecting fungal cells from antifungal agents. It is unclear as how EVs can be transported across the cell wall. **c** Physiological relevance of this study is depicted by a proposed speculative model where EVs from WT cells can rescue stressed fungal cells with defective cell walls

If EVs function to protect yeast cells against the effects of antifungal agents such as caspofungin, it would be expected that part of the cellular response to sub-inhibitory levels of antifungal would be to increase EV release. EVs were isolated from wildtype yeast cells treated with a range of caspofungin concentrations and subjected to protein quantification and NTA. The total protein amount of EVs isolated from caspofungin treated yeast increased compared to non-treated yeast in a concentration-dependent manner (Fig. 7a). These results confirm that part of the cellular response to caspofungin is the increased release of EVs.

## Discussion

The mechanism of EV biogenesis in fungi is poorly understood. In mammalian models, biogenesis of exosomes, a subclass of EVs, is regulated, at least in part, by the ESCRT machinery^10^. The ESCRT machinery is highly conserved throughout eukaryotes and much of the current knowledge pertaining to ESCRT is based on yeast models^36^. It is well established that the ESCRT components are enriched in exosomes secreted by mammalian cells and hence are often used as markers of exosomes^37–39^. However, fungal EVs are depleted of ESCRT proteins as cargo. This study was consistent with previous reports^24^ that yeast EVs are depleted of ESCRT components suggesting that yeast EVs are different to mammalian exosomes or that the ESCRT machinery is not secreted via EVs. Importantly, the amount, size, morphology and proteomic profiles of yeast EVs were altered in strains with selective knockouts of ESCRT components. This was consistent with previous reports where *C. albicans* ESCRT deletion strains produced less EVs than wildtype^11^. Thus, ESCRT components are likely to regulate EV production in yeast, but the mechanism is not the same as in mammals. The lack of ESCRT proteins in yeast EVs points to a fundamental difference in how the ESCRT complexes function to generate the small intraluminal vesicles within MVBs. Current models for mammalian cells indicate that when vesicles are formed in an ESCRT dependent pathway, the ESCRT machinery remains associated with the vesicle. Therefore, the lack of yeast homologues of these proteins in EVs indicates that in a yeast system, the mechanism of vesicle budding from the MVB membrane that is controlled by ESCRT is different to mammalian cells. The data presented here does not dispute the role for ESCRT in the biogenesis of yeast EVs, as strains with deletions in ESCRT components had significant differences in EV production. It merely indicates that the conservation of ESCRT mediated processes may not be as robust as previously thought.

The protective effect of EVs against the antifungal agents caspofungin and NaD1 led to two hypotheses to explain the mechanism of protection (Fig. 7b). These were both different from the role fungal EVs have in protecting *C. albicans* against azole antifungals via secretion of biofilm matrix materials^11^. The first was that EVs were acting as decoys, binding to and sequestering the antifungal agents, preventing them from accessing the fungal cell thereby decreasing their efficacy. The second was that EVs were taken up by the target cells and elicit a response that results in the protection against the antifungal, in this case reinforcement of the cell wall. Based on our observations that labelled EVs are taken up by yeast cells, that the inclusion of a wash step between EV treatment and antifungal treatment does not result in a loss of protection and the enrichment of cell wall biosynthetic enzymes in yeast EVs, we hypothesize that these EVs have a role in cell wall dynamics and exert their protective effect via uptake and remodelling the cell wall. However, a decoy effect of EVs where they can protect the yeast cells by sequestering the antifungals cannot be ignored. Further research is warranted to dissect the precise role of these mechanisms in fungal cell stress response in physiological conditions.

Rescue of the caspofungin sensitive phenotype of *chs3*Δ by treatment with EVs, especially the most prominent rescue with *vps2*∆ and *vps23*∆ EVs which are enriched in glucan and chitin synthases respectively, further supported the role for these vesicles in cell wall biosynthesis and remodelling. The protective effect against caspofungin extended to wild type yeast and was confirmed to be dependent on the protein cargo contained in the EVs and not merely on the presence of membrane-bound vesicles. Conversely, the protective effect of the EVs was also dependent on the integrity of the EVs as disruption of the EV membrane decreased the rescue. Hence the protective effect of EVs requires the packaging of specific cargo into an intact vesicle for secretion and uptake. This discovery of cargo-dependent functional EVs in yeast highlights the immense potential of extending the field of fungal EV research. Further supporting the role for EVs in cell wall dynamics and the response to cell wall damaging agents was the concentration-dependent increase in EV release upon caspofungin treatment. Depletion of cell wall 1,3-β-glucan by deletion of *fks1* also led to increased EV release. The cell wall is often discussed as a barrier to EV release and it is difficult to dissect whether the increase in EV release is the result of increased biogenesis as part of a stress response or merely depleting the cell wall as a barrier to release. Hence, additional research is required to further investigate EVs and stress responses.

Though our study advanced the understanding of fungal EV biology and proposes novel roles for EVs as either decoys or cell wall remodelling components, there are few limitations that need to be addressed by future studies. For instance, the physiological relevance of many EV studies has been unclear (see current issues in EV research^4^) as the stoichiometry of EV release remains elusive. Adding to the challenge, EVs are secreted constitutively in physiological conditions and how such a concentration of EVs specifically relate to a bolus dose of EVs that is often used in vitro in many studies is unclear. Hence, the physiological relevance of the concentration of EVs used in this study need to be examined. Perhaps, co-culture experiments with fungal cells whose EV release can be conditionally regulated would serve as an ideal setup to examine the physiological relevance of EVs. Another limitation relates to the possibility that perturbation of genes in cells may allow for depletion or enrichment of certain EV subtypes (e.g. exosomes). For instance, it is possible that *vps23∆* mutant may secrete less endocytic EVs and hence the isolation procedure may enrich for membrane-derived EVs. Though we employed a 15,000 × *g* centrifugation step to deplete large EVs, it has to be acknowledged that none of the currently available protocols can isolate any one EV subtype to homogeneity^5,40^. Hence, it is possible that our EV preparation may contain some minor population of large EVs. Clearly, additional research is needed to develop efficient tools/reagents to purify EV subtypes to homogeneity and to characterise EVs based on specific markers.

Nevertheless, in a natural environment, intercellular communications through EVs may assist in increasing the survival of yeast cells under conditions that damage the cell walls, such as NaCl stress (Fig. 7c). Demonstration of the uptake and transfer of functional cargos by EVs in fungal cells extends their role beyond host-pathogen interactions into the realm of quorum sensing and interspecies communication. It is fathomable that a population of drug-resistant fungi may be able to transfer resistant enzymes or enzyme products to non-resistant cells to bolster an infection. Whether it is possible for one fungal species to transfer cargo to another via EVs is a question that will need to be addressed in future studies.

## Methods

### Yeast strains and media

The *S. cerevisiae* non-essential gene deletion collection was purchased from OpenBiosystems (Thermo Scientific) and is in the *S. cerevisiae* BY4741 (*MAT*α *his3*Δ*0 leu2*Δ*0 met15*Δ*0 ura3*Δ*0*) background. The mutant strains were retrieved from the deletion collection and compared to the wildtype BY4741. Double mutants were made by amplifying the URA3 gene from the pRS426 plasmid using primers containing 40-bp regions corresponding to the 5’ and 3’ ends of the coding region upstream of the pRS426 binding sequence (bases shown below in italics). The primer pairs were Vps23F (ATCTTAACGGCCAAGAAAAGAGAGAGAGTGAAGAGCAACG*CTGTGCGGTATTTCACACCG*) and Vps23R (ATATTTTTTATGGCACTTCGGCGATGCGAAAGAAAGTGAG*AGATTGTACTGAGAGTGCAC*). PCR products were purified using a Wizard PCR clean-up kit (Promega) and transformed into yeast cells via electroporation. The mutant colonies were selected on synthetic defined medium without uracil (SD-Ura) (0.67% yeast nitrogen base without amino acids [Sigma], 0.077% uracil dropout (Ura DO) supplement [Clontech]) agar plates. Overnight cultures for all *S. cerevisiae* experiments were grown in YPD medium (1% yeast extract, 2% peptone, 2% dextrose). All mutants including those retrieved from the library were confirmed by PCR genotyping^41,42^.

### EV isolation

Isolation of EVs was performed as described previously with some modifications^24,43–45^. Overnight cultures of *S. cerevisiae* were diluted to an OD_600_ of 0.2 with YPD medium. Cultures were then incubated for 18 h at 30 °C with shaking (130 rpm). For EV isolation, cells and debris were removed by differential centrifugation at 4000 × *g* for 15 min and 15,000 × *g* for 15 min. Supernatants were collected and ultracentrifuged at 100,000 × *g* for 1 h at 4 °C. Pellets were collected and washed once with 1 × phosphate-buffered saline (PBS). The resulting EV pellets were resuspended in 1 × PBS and stored at −80 °C. For isolation of larger vesicles when required, pellets from 15,000 × *g* centrifugations were collected and washed once with 1 × PBS. The resulting pellets were resuspended in 1 × PBS and stored at −80 °C. For EV isolation from caspofungin treated yeast, overnight cultures of *S. cerevisiae* were diluted to an OD_600_ of 0.4 with YPD medium, incubated for 2 h at 30 °C with shaking until reaching an OD_600_ of 0.86, treated with caspofungin and incubated for 14 h at 30 °C with shaking. Cultures were then subjected to EV isolation. For EV isolation from heat killed yeast, cell pellets from 4000 × *g* centrifugation were also collected. Cell pellets were resuspended in YPD medium and heated at 70 °C for 2.5 h with shaking (750 rpm). Heat killed cells were then resuspended in same volume of YPD medium as the previous culture, incubated for 18 h at 30 °C with shaking again, and proceeded to the same process of EV collection.

### Transmission electron microscopy (TEM)

Microscopy was performed as described previously^46^. EV samples (0.2 μg/μL each) were examined with a JEM-2010 transmission electron microscope (JEOL, 100 kV) or Tecnai TF30 transmission electron microscope (FEI, 300 kV). Preparations were fixed to 400 mesh carbon-layered copper grids for up to 2 min. Surplus material was drained by blotting, followed by negative staining of samples with 10 μL of uranyl acetate solution (2% w/v; Electron Microscopy Services).

### Nanoparticle tracking analysis (NTA)

Size distributions of EV samples were analysed with NanoSight NS300. Samples were diluted in water (Milli-Q) and injected using a syringe pump with a flow rate of 50, and 1 min videos were taken. Data were obtained in triplicate and was analysed using NTA 3.2 Dev Build 3.2.16 with the auto-analysis settings.

### Protein quantification

The SYPRO Ruby staining method was used to quantify protein amounts. Proteins were separated using SDS-PAGE, and Benchmark ladder (Life Technologies) was used as a protein standard for the following quantifications. The SDS-PAGE gels were immersed in fixation solution (40% (v/v) methanol, 10% (v/v) acetic acid) for 30 min with shaking. Gels were kept overnight in SYPRO Ruby fluorescent dye stain (Molecular Probes) and were then washed with destain solution (7.5% (v/v) acetic acid, 20% (v/v) methanol) for 30 min. Fluorescent signals were visualized using Typhoon Trio™ scanner (GE Healthcare) or Typhoon™ FLA 7000 scanner (GE Healthcare). Protein amounts were quantified by densitometric analysis with ImageQuant™ software (GE Healthcare).

### SDS-PAGE and tryptic digestion

Proteins (30 µg) were separated by SDS-PAGE and visualized with Coomassie staining (Bio-Rad). Gel lanes were excised into sections (10 for each lane) and subjected to in-gel reduction, alkylation and trypsination as described previously with modifications^43,44,47^. Briefly, proteins in the gel sections were reduced with 10 mM DTT (Bio-Rad) for 30 min at 55 °C, alkylated for 30 min with 25 mM iodoacetamide (Sigma), and then digested overnight at 37 °C with 750 ng of sequencing grade trypsin (Promega). Digestion products were extracted with 50% (v/v) acetonitrile and 0.1% trifluoroacetic acid and were then analysed by liquid chromatography-mass spectrometry (LC-MS/MS).

### LC-MS/MS

Extracted tryptic peptides from each gel band were concentrated to ~10 μL by centrifugal lyophilisation and analysed by LC-MS/MS using LTQ Orbitrap Elite and Fusion Lumos mass spectrometer (Thermo Scientific), both fitted with nanoflow reversed-phase-HPLC (Ultimate 3000 RSLC, Dionex). The nano-HPLC system was equipped with an Acclaim Pepmap nano-trap column (Dionex—C18, 100 Å, 75 μm × 2 cm) and an Acclaim Pepmap RSLC analytical column (Dionex—C18, 100 Å, 75 μm × 15 cm). Typically, for each LC-MS/MS experiment, 1 μL of the peptide mix was loaded onto the enrichment (trap) column at an isocratic flow of 5 μL/min of 3% CH_3_CN containing 0.1% formic acid for 5 min before the enrichment column is switched in-line with the analytical column. The eluents used for the LC were 0.1% v/v formic acid (solvent A) and 100% CH_3_CN/0.1% formic acid v/v. The gradient used was 3% B to 25% B for 23 min, 25% B to 40% B in 2 min, 40% B to 85% B in 2 min and maintained at 85% B for 2 min before equilibration for 10 min at 3% B prior to the next injection. All spectra were acquired in positive mode with full scan MS spectra scanning from m/z 300–1650 in the FT mode at 240,000 resolution after accumulating to a target value of 1.00e^6^ with maximum accumulation time of 200 ms. Lockmass of 445.12003 *m/z* was used. For MSMS on the Lumos orbitrap, the “top speed” acquisition method mode (3 s cycle time) on the most intense precursor was used whereby peptide ions with charge states ≥2 were isolated with isolation window of 1.6 *m/z* and fragmented with low energy CID using normalized collision energy of 35 and activation Q of 0.25. For MSMS on the Elite orbitrap, the 20 most intense peptide ions with minimum target value of 2000 and charge states ≥2 were isolated with isolation window of 1.6 *m/z* and fragmented by low energy CID with normalized collision energy of 30 and activation Q of 0.25. Dynamic exclusion settings of 2 repeat counts over 30 s and exclusion duration of 45 s was applied.

### Database searching and protein identification

Mascot Generic File Format (MGF) files were generated using MSConvert with the parameter of peak picking set. X!Tandem VENGEANCE (2015.12.15) was then used to search the MGF files against a target and decoy Yeast RefSeq protein database. Search parameters used were: fixed modification (carboamidomethylation of cysteine; +57Da), variable modifications (oxidation of methionine; +16 Da and N-terminal acetylation; +42 Da), three missed tryptic cleavages, 20 ppm peptide mass tolerance and 0.6Da fragment ion mass tolerance. Protein identifications were shortlisted to obtain a master list with less than 1% false discovery rate^43^.

### Label-free spectral counting

The relative protein abundance between the samples was obtained by estimating the ratio of normalized spectral counts (RSc) as described previously^44,48^.

$$\begin{array}{l}{\mathrm{RSc}}\,{\mathrm{for}}\,{\mathrm{protein}}\,{\mathrm{A}}\\ {\mathrm{ = }}\, \left[ {\left( {{\mathrm{sY + c}}} \right)\left( {{\mathrm{TX - sX + c}}} \right){\mathrm{/}}\left( {{\mathrm{sX + c}}} \right)\left( {{\mathrm{TY - sY + c}}} \right)} \right]\end{array}$$

Where s is the significant MS/MS spectra for protein A, T is the total number of significant MS/MS spectra in the sample, c is the correction factor set to 1.25, and X or Y are the EV samples. When RSc is less than 1, the negative inverse RSc value was used.

### *S. cerevisiae* growth and death assays

Assays were performed as described previously with modifications^41,49^. For growth assays, cells were grown in YPD overnight and diluted to an OD_600_ of 0.01. Diluted cells (90 µL) were added to the wells of a 96-well plate along with 10 µL of the test drugs. Growth of cells was monitored by measuring absorbance at 595 nm in a SpectraMAX M5e plate reader (Molecular Devices), using a 96-well scan. Measurements were taken at *t* = 0 and *t* = 18 h during incubation at 30 °C. For death assays, cells were grown in YPD overnight and diluted to an OD_600_ of 0.5. Diluted cells (90 µL) were added to 96-well plate along with 10 µL of the test drugs. Plates were incubated for 1 h at 30 °C. Death of cells was measured by spot and plating assays.

### Time-lapse live imaging

Cells diluted to 2 × 10^6^ cells/mL (OD_600_ of 1 is ~3 × 10^7^ cells/mL) from YPD overnight cultures were used for live imaging. Cells with or without H_2_O_2_ (Merck) treatment were loaded into four-well Nunc Lab-Tek II chambered cover glass. Imaging was performed as described previously with modifications^50^. Time-lapse DIC microscopy was performed at 30 °C using the Zeiss Spinning Disk Confocal with a 63 oil-immersion objective. For most of the experiments, samples were imaged every 3 min for 3.5 h. Image processing and data analysis were performed using the ZEN imaging software (Zeiss, Germany).

### Uptake assays

To stain EVs, 100 µg of EVs was incubated with 2 µM of lipophilic membrane dye PKH67 (Sigma® Life Science’s cell linker kit) for 3–5 min at room temperature. The labelling reaction was stopped by addition of 1% bovine serum albumin (BSA). Stained EVs were collected on a 100 kDa filter, washed three times with 5 mL 1 × PBS, suspended in 1 mL of 1 × PBS and centrifuged at 120,000 × *g* for 50 min at 4 °C. The resulting pellet of stained EVs was resuspended in 1 × PBS. Cells were grown in YPD overnight. Cells (3 × 10^6^) were added into a 1.5 mL microfuge tube along with stained EVs and were incubated for different time points at 30 °C with shaking. The resulting cells were washed once with 1 mL 1 × PBS and checked by flow cytometry (BD FACSCantoTM II) and confocal microscopy (LSM 780, Zeiss) as described previously^37^. Image processing was performed with the ZEN imaging software (Zeiss, Germany).

### Functional uptake assays

Cells were grown in YPD overnight and diluted to an OD600 of 0.1. EVs were diluted to a concentration of 1 µg/µL with 1 × PBS. 10 µL of diluted cells, 10 µL of diluted EVs and 80 µL of medium (YPD medium for caspofungin [Sigma] assays, half-strength PDB medium for NaD1 assays) (half-strength PDB medium: 1.2% potato dextrose broth powder [BD Difco]) were added together into 1.5 mL Eppendorf tubes and incubated for 30 min at 30 °C with shaking. Tubes were then incubated for another 30 min at 30 °C without shaking. When needed, cells were washed once with medium before drug treatments, 90 µL of cells was then added into new 1.5 mL Eppendorf tubes along with 10 µL of caspofungin or NaD1 at 10× the final concentration and were incubated for 2 h at 30 °C. Cell survival was measured by serial dilution spot assays and plating for colony counts as described below. NaD1 used in the assays was purified from plant sources as described previously^51^.

### EV sonication

EVs were diluted with YPD medium in 1.5 mL Eppendorf tubes and sonicated using Vibra-Cell™ (Sonics & Materials) sonicator and Microtip probe (QSonica). Ten cycles (5 min of 100 output control, 1 min interval) of sonication were performed for samples in an ice-water bath. For confirming the outcome of sonication, samples were ultracentrifuged at 100,000 × *g* for 1 h at 4 °C. Pellets were then resuspended with 1 × PBS and subjected to NTA. For functional uptake assays, 10 µL of cells with an OD_600_ of 0.1 was added in 90 µL of sonicated YPD solution with 5 µg of EVs, then followed by incubations and caspofungin treatments as described earlier.

### Spot and plating assays

Assays were performed as described previously with modifications^41,42,49^. For death assays, cells were serially diluted (5×) four times in YPD medium in the wells of a 96-well plate. For functional uptake assay, cells were washed once with YPD medium before the serial dilution. 4 µL of each dilution was spotted onto YPD agar medium. Plates were incubated for 24 h at 30 °C and images of the resulting colony growth were taken. To quantify the results, for death assays, cells were 1:2000 diluted with YPD medium. For functional uptake assays, cells were diluted 1:25. For co-culture assays, cells were diluted 1:250 and 50 µL of diluted cells were plated on YPD agar medium. Plates were incubated for 24 h at 30 °C and left overnight at room temperature. The resulting colonies were imaged and counted.

### Statistics and reproducibility

Statistical analysis was performed with *t*-test or Chi-square test, and differences were considered as significant while *P-*value <0.05. Data are presented as mean ± standard deviation (SD) or standard error of the mean (SEM). The results included in this manuscript were obtained from at least 3 independent experiments and were reproducible.

### Reporting summary

Further information on research design is available in the Nature Research Reporting Summary linked to this article.

## Supplementary information

Supplementary Figures

Description of Additional Supplementary Files

Reporting Summary

Supplementary Data 1

Supplementary Data 2

Supplementary Data 3

## Data Availability

All data analysed during this study are included in this published article and its Supplementary Information files. The lists of identified EV proteins are available as Supplementary Data 1 and 2. Proteomics data of EVs are also available through Vesiclepedia^29,30^ database: [http://microvesicles.org/browse_results?org_name=Saccharomyces%20cerevisiae&cont_type=&tissue=&gene_symbol=&ves_type=]. The source data behind the graphs are available as Supplementary Data 3. All other data are available from the corresponding author on reasonable request.
