# Supplementary material for: Extracellular vesicles secreted by Saccharomyces cerevisiae are involved in cell wall remodelling
Source: Commun Biol. 2019 Aug 9;2:305. doi: 10.1038/s42003-019-0538-8 (PMC6688994; doi:10.1038/s42003-019-0538-8)
Supplement: Supplementary file 1 — Supplementary Figures [file 42003_2019_538_MOESM1_ESM.pdf]

Supplementary Figure 1

a

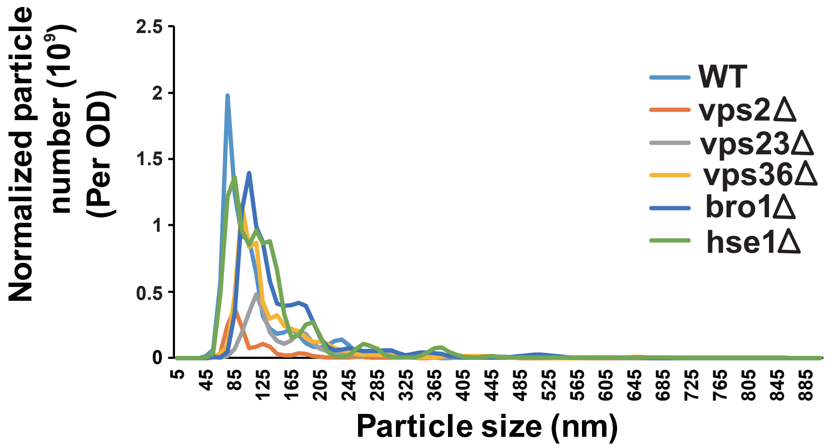

b

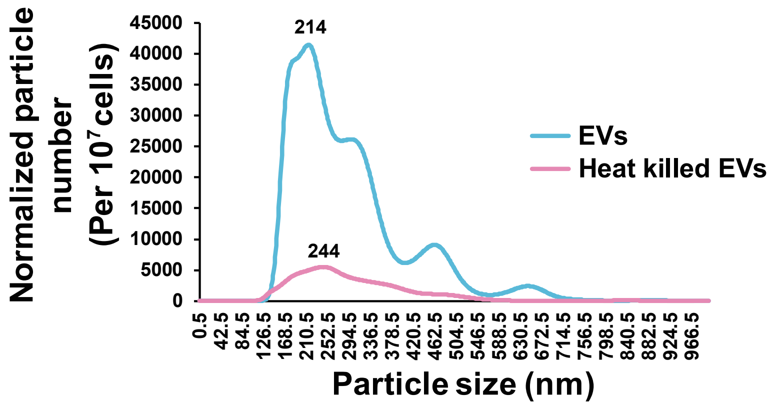

Supplementary Figure 1

NTA analysis of EVs

(**a**) NTA of EVs depicts that particle numbers of EVs isolated from *vps2*Δ, *vps23*Δ and *vps36*Δ were less than EVs from WT (normalized to OD, n = 3). (**b**) NTA shows that particle number of EVs isolated from heat killed WT yeast is lower than EVs isolated from same amount of live WT cells.

Supplementary Figure 2

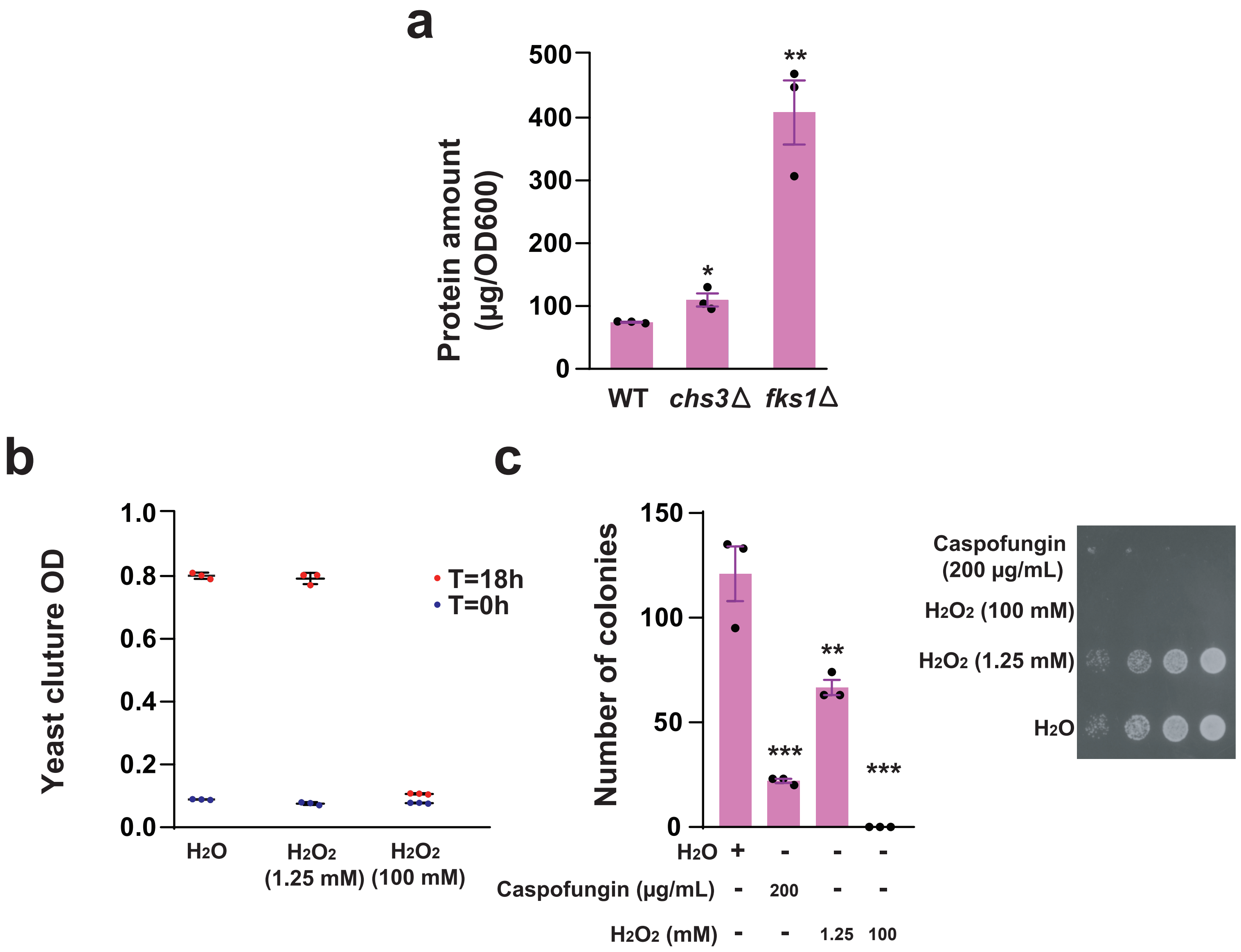

Supplementary Figure 2

Effect of EV subtype inducers and inhibitors on yeast cell growth

(**a**) Total protein amounts in EVs from *fks1*Δ and *chs3*Δ cells were significantly higher than protein levels in WT EVs (normalised to OD) (* denotes $P \leq 0.05$, ** denotes $P \leq 0.01$, *** denotes $P \leq 0.001$ as determined by two-tailed t-test; Error bar = ±SEM, $n = 3$). (**b-d**) Prior to EV isolation, tolerance tests for H_2O_2 was performed to determine suitable concentrations for treating yeast cultures (* denotes $P \leq 0.05$, ** denotes $P \leq 0.01$, *** denotes $P \leq 0.001$ as determined by two-tailed t-test; Error bar = ±SD, $n = 3$).
