# Supplementary material for: Extracellular vesicles secreted by Saccharomyces cerevisiae are involved in cell wall remodelling
Source: Commun Biol. 2019 Aug 9;2:305. doi: 10.1038/s42003-019-0538-8 (PMC6688994; doi:10.1038/s42003-019-0538-8)
Supplement: Supplementary file 2 — Description of Additional Supplementary Files [file 42003_2019_538_MOESM2_ESM.docx]

**Description of Additional Supplementary Files**

**File Name**: Supplementary Data 1

**Description**: The document contains the list of proteins identified in yeast EVs

**File Name**: Supplementary Data 2

**Description**: The document contains the list of proteins identified in yeast EV subtypes

**File Name**: Supplementary Data 3

**Description**: This document contains all the raw data used to plot the figures
